# The Effects of Nucleating Agents and Processing on the Crystallization and Mechanical Properties of Polylactic Acid: A Review

**DOI:** 10.3390/mi15060776

**Published:** 2024-06-12

**Authors:** Peng Gao, Davide Masato

**Affiliations:** 1 Department of Plastics Engineering, University of Massachusetts Lowell, Lowell, MA 18015, USA; 2 Polymer Materials Engineering, Department of Engineering and Design, Western Washington University, Bellingham, WA 98225, USA

**Keywords:** polylactic acid, crystallization, mechanical properties, nucleating agents, sustainability, shear-induced processing technology

## Abstract

Polylactic acid (PLA) is a biobased, biodegradable, non-toxic polymer widely considered for replacing traditional petroleum-based polymer materials. Being a semi-crystalline material, PLA has great potential in many fields, such as medical implants, drug delivery systems, etc. However, the slow crystallization rate of PLA limited the application and efficient fabrication of highly crystallized PLA products. This review paper investigated and summarized the influence of formulation, compounding, and processing on PLA’s crystallization behaviors and mechanical performances. The paper reviewed the literature from different studies regarding the impact of these factors on critical crystallization parameters, such as the degree of crystallinity, crystallization rate, crystalline morphology, and mechanical properties, such as tensile strength, modulus, elongation, and impact resistance. Understanding the impact of the factors on crystallization and mechanical properties is critical for PLA processing technology innovations to meet the requirements of various applications of PLA.

## 1. Introduction

Biobased polymers have attracted significant interest in academic research and industry applications in the last 15–20 years. Driven by their sustainability benefits, many industries have introduced biobased materials to replace petroleum-based materials for the application [1]. These applications can be found almost everywhere in our daily lives, including medical devices [2], rigid packaging [3], and agriculture [4].

One of the defining features of biobased materials is their derivation from renewable resources, such as plants, agricultural residues, and algae. This feature distinguishes them from fossil-based materials, which rely on non-renewable petroleum resources [5,6,7]. One of the most compelling aspects of biobased materials is that they can reduce environmental impact and carbon footprint throughout the lifecycle [8]. The production and decomposition of biobased materials typically result in lower greenhouse gas emissions than traditional petroleum-based materials [9,10]. Reducing greenhouse gas emissions has been a long-term goal for the global industry. For industries such as flexible packaging, this reduction in greenhouse gas emissions aligns with global efforts to combat climate change and reduce the carbon footprint associated with various industries.

Furthermore, biobased materials often contain fewer toxic additives and chemicals than conventional materials. This inherent characteristic diminishes the pollution and health risks associated with these materials’ manufacturing, use, and disposal. As a result, they are seen as a safer and more sustainable choice for a wide range of applications, especially those that come into direct contact with humans or the environment [11,12,13].

Biobased polymers play a significant role in circular economy models, where products should be designed considering reuse, recycling, or composting at the end of life [7,14,15]. In addition, some biobased materials are also biodegradable. Biodegradable materials are continuously recycled or returned to nature without causing harm by composting and generating H_2_O and CO_2_. This minimizes waste generation, extends the lifespan of materials, and contributes to a more sustainable consumption pattern.

Among the various biobased materials, polylactic acid (PLA) is one of the most promising and well-studied materials. PLA is a biodegradable, biocompatible, and biobased thermoplastic polymer derived from renewable plant sources. The monomer lactic acid is derived using the bacterial fermentation of corn, potatoes, sugarcane, and other biomass. PLA exhibits excellent biodegradability and compostability [16]. It is widely used in various applications, from food packaging to 3D printing, and it has gained popularity as a sustainable alternative to traditional plastics. PLA’s versatility and its environmentally friendly properties make it a key player in the transition toward more sustainable material choices in the 21st century.

## 2. Overview of Polylactic Acid (PLA)

**Figure 1:**
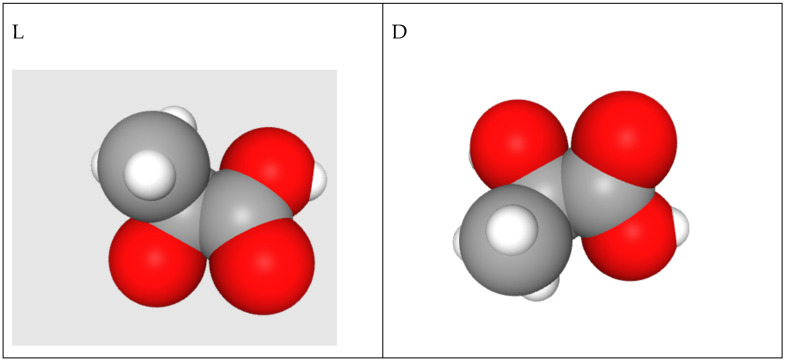
Atomic chemical structure of L- and D-lactic acid (captured from https://pubchem.ncbi.nlm.nih.gov/ accessed on 10 March 2024).

For several compelling reasons, polylactic acid (PLA) holds immense promise as a biopolymer. First, its monomers can be derived from non-toxic and renewable feedstock sources, making it environmentally friendly [16,17]. Additionally, PLA is naturally occurring as lactic acid (2-hydroxypropionic acid), a chiral molecule consisting of two enantiomers: L-lactic acid and D-lactic acid (c.f. Figure 1) [18]. This inherent chirality leads to the formation of various stereoisomers of PLA, such as poly(L-lactide) (PLLA), poly(D-lactide) (PDLA), and poly(DL-lactide) (PDLLA).

Lactic acid, the repeating unit of PLA, is produced through the fermentation of sugars obtained from renewable resources, such as sugarcane or corn starch. Consequently, PLA emerges as an eco-friendly material with excellent properties for applications within the human body. Additionally, PLA is recognized as “generally recognized as safe” (GRAS) by the United States Food and Drug Administration (FDA). Nowadays, PLA is used in various food packaging applications [19].

The FDA’s approval of PLA and its blends for medical applications has encouraged research and innovation in medical applications, including implants, drug delivery devices, bone fixtures, and more. Over the past two decades, the utilization of large-scale industrial lactic acid-based polymers has expanded beyond medical applications to embrace a broader range of uses. PLA is now used in the construction, electronic, agricultural, and automotive industries. This trend underscores PLA’s versatility and growing significance in materials science and industrial applications [20,21,22].

One of the primary challenges faced during the early stages of PLA synthesis was to achieve higher molecular weight (Mw). Higher Mw is critical for improving the performances of the polymer materials, such as mechanical strength, impact resistance, chemical resistance, and durability [23,24,25]. The breakthrough came with ring-opening polymerization of lactide, a cyclic dimer of lactic acid [26,27]. By opening the lactide ring and allowing it to polymerize, scientists could synthesize high-molecular-weight PLA. This advancement significantly improved PLA’s overall properties and made PLA suitable for a broader range of applications.

The synthesis technology of PLA can be categorized as follows [17]. Polycondensation is one of the most traditional techniques to fabricate PLA. This method involves the direct condensation of lactic acid molecules to form PLA. It is still employed in specific applications, especially for producing low-Mw PLA. The ring-opening polymerization of lactide remains one of the most widely used methods for PLA production nowadays. It involves the controlled opening of the lactide ring, which undergoes polymerization to form PLA. This method allows for precise control over molecular weight and properties. Manufacturers can achieve specific molecular weight and Mw distribution in the final product by controlling the ring-opening rate. Azeotropic dehydration involves more direct routes to PLA synthesis. It uses catalysts with carefully controlled conditions. Azeotropic dehydration, for instance, consists of removing water as an azeotrope with a suitable solvent, promoting the polymerization of lactic acid. Lastly, one of the most recent techniques is enzymatic polymerization. Enzymatic polymerization methods leverage biological catalysts to facilitate PLA polymerization. It is considered eco-friendly and valuable for specific applications.

Compared to other biopolymers, PLA has numerous advantages [3]. PLA can be produced from renewable resources, such as corn, wheat, or rice [28,29]. During photosynthesis, plants absorb CO_2_ from the atmosphere and convert it into organic compounds, primarily sugars and starches, using sunlight as energy. This natural process helps to sequester CO_2_ from the atmosphere, reducing the concentration of this greenhouse gas. PLA is derived from plants grown for its synthesis; hence, the cultivation of these crops actively contributes to carbon dioxide consumption. Unlike traditional plastics resourced from fossil fuels and natural gases, extracting and processing fossil fuels release CO_2_ and other greenhouse gases into the atmosphere, contributing to global warming and climate change. Furthermore, the manufacturing process for PLA typically requires less energy and generates fewer greenhouse gas emissions, reducing its overall carbon footprint [30]. Not only biobased, PLA’s most appealing aspect, especially in biomedical applications, is its biocompatibility. Biocompatible materials do not produce toxic effects in local tissues, especially in human bodies. Their degradation products should not interfere with tissue healing. PLA undergoes hydrolysis in living organisms, including the human body, converting into non-toxic α-hydroxy acids, which are incorporated into the tricarboxylic acid cycle and excreted. This makes PLA an excellent choice for biomedical applications. It has received FDA approval for direct contact with biological fluids [31]. PLA also exhibits excellent thermal processibility compared to other biopolymers, such as poly(hydroxyl alkanoate) (PHA), poly(ethylene glycol) (PEG), and poly(γ-caprolactone) (PCL). It can be processed using various methods, including injection molding, film extrusion, blow molding, thermoforming, fiber spinning, and film forming [16]. Lastly, PLA production is more energy-efficient, requiring 25–55% less energy than petroleum-based polymers with similar mechanical and thermal properties. Future estimates suggest it can be reduced to less than 10% [31]. Lower energy consumption aligns with the environmental goals of using a biobased material and potentially lowers production costs.

However, despite these positive attributes, PLA has its limitations. Product performance-wise, PLA is brittle, with less than 10% elongation at break under tensile testing [31,32]. While its tensile strength and elastic modulus are comparable to poly(ethylene terephthalate) (PET), it allows PLA to replace PET for applications such as flexible packaging and single-use utensils. However, the lack of toughness limits its use in applications under higher stress levels and loads, such as screws and fracture fixation plates [31]. PLA has a methyl group as the side-chain group connecting to the α carbon. The lack of reactive side-chain groups makes the processing easy for PLA; however, it reduces cross-linking and thermal stability. This limits the application of PLA under high-temperature conditions. For the same reason, the surface properties, such as adhesion and compatibility with other polymers, are also limited. Lastly, the reactive side-chain groups will promote the biodegradability of the polymer by incorporating hydrolysable or oxidizable groups. PLA’s lack of such groups will make the material more resistant to degradation, which is not ideal for environmental sustainability [33,34,35]. Finally, the slow crystallization rate and the relatively low degree of crystallization are notable characteristics of PLA. The low degree of crystallinity leads to the lack of high stiffness, strength, heat deflection temperature, and chemical resistance [36,37,38]. Moreover, the slow crystallization rate of PLA limits the fabrication of high-crystalline PLA products. In most applications, manufactured products are characterized by amorphous morphology. Post-fabrication techniques, such as annealing, are implemented if a high-crystallinity PLA product is required. However, this leads to a longer fabrication time, the need for high-temperature equipment, and possible geometrical instability for 3D complex products. 

Due to the lack of mechanical properties and low degree of crystallization of the PLA products, amorphous PLA is mainly used for single-use packaging. These applications leverage PLA’s ability to decompose rapidly in landfill or compost environments. Commercialized processing methods for the packaging industry include film production, blow molding, and paper coating to enhance water resistance. High-crystallinity PLA finds applications in many fields, such as medical implants, sutures, automotive parts, and sporting goods (e.g., bicycle helmets and snowboards), where strength, durability, and biodegradation are critical. However, due to the slow crystallization rate of PLA, the commercialization of these applications is still limited. 

This paper provides a comprehensive and critical review of the existing literature and research on controlling PLA’s crystallinity and crystallization behavior. Different approaches to material modification (polymer blending, nucleating agent compounding) and process innovation (annealing, cooling techniques, shear control during fabrication) to enhance the crystallization behavior of PLA are studied and summarized. Various crystallization properties are considered, such as the degree of crystallization, crystallization rate, nucleating density, and morphology.

## 3. PLA Crystallization

The crystallization behavior of PLA is a crucial aspect because it significantly influences the properties and performances of the product. PLA is a semi-crystalline polymer with a crystallization process similar to other polymers. Crystallization in PLA typically begins with nucleation, where molecular chains start to organize into crystalline structures [39]. The nucleation of PLA can occur spontaneously or be induced by the addition of nucleating agents or by controlling processing conditions [40]. There are two primary ways nucleation can occur. Homogeneous nucleation is the spontaneous formation of tiny crystalline domains within the amorphous polymer matrix. This happens when PLA chains experience local fluctuations and modifications in chain alignment. The movement is commonly observed at the molecular level with the formation of small crystalline nuclei. Homogeneous nucleation is rare for PLA due to the lack of reaction side-chain groups in its chemical structure. Some studies suggested that homogeneous nucleation could occur at a slow cooling rate under quiescent conditions for PLLA materials [41,42].

- Heterogeneous nucleation, initiated by nucleating agents, is more commonly observed in PLA. Nucleating agents provide sites where PLA chains can organize and form crystalline nuclei. These agents can be inorganic materials, such as talc; organic compounds, such as sorbitol; or a specific PLLA-PDLA matrix known as stereocomplex PLA. Nucleating agents in the PLA polymer matrix serve as crystallization catalysts, reducing the energy barrier for nucleation [41,43]. Once nucleation sites are formed during the nucleation step, crystalline domains grow in the polymer matrix. PLA exhibits more than one form or phase of crystalline structures. The most common structure is the α-form. These crystalline domains consist of regularly arranged polymer chains, which increase the PLA strength and stiffness compared to amorphous regions. Depending on the nucleating agent added, processing techniques, and crystallization conditions, PLA can present four distinct crystalline forms, as summarized in Table 1.

**Table 1:** Common crystalline forms of PLA.

Crystalline Form	Crystal System	Cell Parameters					
		a (nm)	b (nm)	c (nm)	α (°)	β (°)	γ (°)
α [43]	Orthorhombic	10.05	0.61	2.88	90	90	90
α and α′ [44]	Pseudo-orthorhombic	1.07	0.645	2.78	90	90	90
β [45]	Orthorhombic	1.031	1.821	0.9	90	90	90
β [46]	Trigonal	1.052	1.052	0.88	90	90	90
γ [47]	Orthorhombic	0.995	0.625	0.88	90	90	90
SC [48]	Triclinic	0.916	0.916	0.87	109	109	110
SC [49]	Triclinic	1.498	1.498	0.87	90	90	120

Studies about the morphology and crystalline structures can be traced back to 1968 when De Santis et al. observed poly (S-lactic acid) crystalline structures using X-ray fiber photographs. The polymer sample was annealed at 120 °C for crystallization and stretched before observation. The observed intensities fitted the unit cell with a pseudo-orthorhombic space group with two chains in the unit cell. An α-helix type structure was introduced for PLA for the first time in the article, along with observations of a less stable crystalline domain when PLA was etched in a solvent [45]. Kobayashi et al. confirmed the observation of both crystalline forms in 1995 and reported larger crystalline domains for α-phase crystals. The transition from the unstable form to the α-phase was also observed. The unstable phase was named α’ afterwards [44]. PLA’s most prevalent crystalline structure, the α-form, typically crystallizes from either the melt or solvents under quiescent conditions. The less ordered α′ crystal forms from the melt or solvents but at lower temperatures (<120 °C) [50,51,52]. 

The β-form, on the other hand, tends to develop under conditions involving high shear forces and elevated temperatures. Puiggali et al. reported a frustrated structure (trigonal unit-cell, three helices per cell) for PLLA. The structure can accommodate a random orientation of chains when exposed to rapid crystallization and stretching. The structure was named the β-form and was further studied by the same group via crystallization on hexamethylbenzene (HMB) [46,47,48]. The γ crystal is primarily obtained using a hexamethylbenzene substrate through epitaxial growth and is rarely observed under other conditions. It was also reported by the same group that developed the study of β-form crystalline structures [48].

Recently, a novel crystal form known as stereocomplex crystals or SC-crystals has garnered significant attention. What sets the SC-crystal apart is its considerably higher melting point of 230 °C [53], which exceeds the melting points of the other PLA crystal forms by 50–70 °C [49,54,55,56]. Shao et al. reported that SC-crystal formation was only observed when L-lactic acid and D-lactic acid monomers were polymerized together. This results in a polymer chain with alternating L and D chirality, which allows for forming a tightly packed, highly ordered crystalline structure. These crystals exhibit enhanced thermal and mechanical properties compared to the amorphous or semi-crystalline forms of PLA. The melting point was observed to increase and then decrease as the molecular weight of PLLA changed from 4 to 100 kg/mol. The samples prepared with solvent casting and dried at 50 °C showed a melting point at 230 °C when the molecular weight was between 23 and 50 kg/mol for both PLLA and PDLA. The highest melting point was measured at 249.9 °C after annealing at 120 °C to enhance crystalline growth [53].

PLA chains align and organize along the crystal lattice structure during the crystalline domain growth step. As more PLA polymer chains join the crystalline regions, the crystalline domains grow and become more ordered. The mobility of PLA chains in the amorphous regions decreases since the free volume decreases as the volume is occupied by the highly packed crystalline regions [16]. At the ideal conditions for crystallization, the PLA molecules have enough energy for movement to form orderly packed crystalline regions. Conversely, in the amorphous regions, the molecular movement requires higher energy. This energy barrier represents the minimum energy necessary to arrange the polymer chains into the regular, ordered structure of the crystal lattice. Nucleating agents and appropriate processing conditions can lower this energy barrier, facilitating crystal growth [57,58,59,60].

**Figure 2:**
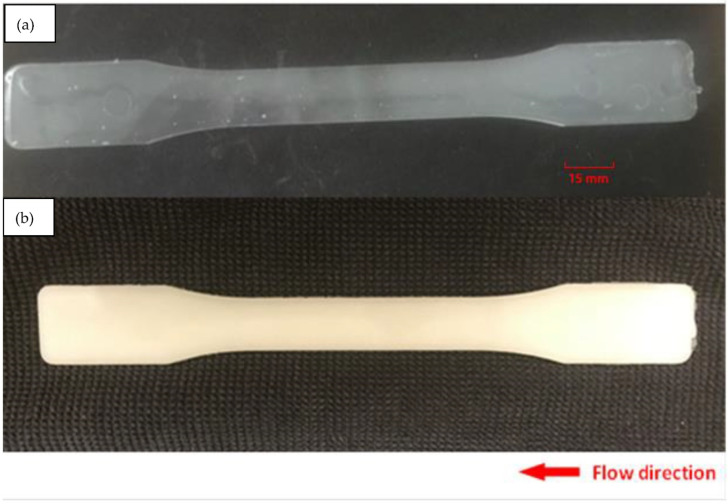
Injection molded sample: (**a**) neat PLA, (**b**) PLA–OA blend at 90 °C mold temperature [73].

The crystallization behavior of PLA is important in determining various properties and influencing its suitability for different applications. The degree of crystallinity in PLA strongly correlates with its mechanical properties, such as tensile strength, impact resistance, and brittleness, which is critical for load-bearing applications. Commonly speaking, higher crystallinity was reported to increase PLA stiffness and strength [9,61,62,63]. The degree of crystallinity also affects PLA’s heat resistance and thermal stability. Highly crystalline PLA has a higher melting point, making it more resistant to deformation at elevated temperatures. This property is essential for high-temperature applications [64,65,66,67]. PLA’s crystallinity also impacts its transparency. Amorphous regions are more transparent, while crystalline regions are opaque (c.f.: Figure 2). This property is relevant in applications such as packaging and other see-through products, where transparency is desirable [65,68,69]. Finally, the rate of biodegradation can be influenced by crystallinity. Crystalline regions tend to degrade more slowly, while amorphous regions are more susceptible to hydrolytic degradation. This balance is essential in applications such as biodegradable packaging and medical applications [34,70,71,72,73].

Different approaches have been introduced to affect the degree of crystallization and the crystallization behavior of PLA and its compounds. They can be sorted into the following categories: material innovation and processing optimization.

## 4. Material Innovations in PLA

Recent advancements in PLA materials have seen innovative approaches to enhancing their crystallinity and properties by introducing nucleation additives, reinforcing them with nanomaterials, and developing biocompatible and biodegradable variants.

One of the significant challenges of obtaining PLA with a high degree of crystallinity is that the nucleation of PLA is rare and the formation of initial nuclei is limited. Many studies have reported a variety of physical nucleating agents for PLA.

### 4.1. Inorganic Additives

#### 4.1.1. Mineral Materials

Due to its low costs and effectiveness on reinforcement, talc has been established as a popular choice for enhancing PLA crystallization [74,75,76,77,78,79,80,81,82,83,84]. Kolstad et al. reported that 6% of talc increased the nucleation density of PLLA by 500 times, resulting in a 7-fold decrease in crystallization half-time at 100–120 °C [76]. In another study by Battegazzore et al., talc’s effect at different concentrations was studied [80]. The authors reported that the degree of crystallinity increased from 7.8% for neat PLA to 28.3% for PLA–5%talc at 90 °C and from 17.3% for neat PLA to 36.9% for PLA–15%talc for a different grade of PLA. Additionally, the crystallization kinetics were enhanced by the addition of talc. The crystallization behaviors were mainly affected by the concertation and crystallization temperatures. The addition of talc reduced the activation energy for nucleation and created nucleation sites. The morphology of the PLA–talc crystals exhibits an epitaxial growth into a lamellar structure. Other mineral-based materials, such as mica [85], kaolinite [86,87], layered double hydroxides (LDHs) [88,89,90,91], and vermiculite (VMT) [92,93,94], can also serve as nucleating agents for PLA and affect the nucleation density and crystallization rates. 

#### 4.1.2. Nanoparticles

Various nanoparticles, such as nano clay, silica nanoparticles, and other inorganic nano-scale materials with high surface areas, can be effective inorganic nucleating agents for PLA. These nanoparticles provide numerous nucleation sites due to their small size and large surface area. They reduce the energy barrier for nucleation and promote the formation of smaller and more uniformly dispersed crystalline structures in PLA. Due to its layered mineral silicates, nano clay has received increasing attention from industry and academia [95,96,97,98,99,100]. Day et al. reported that a nanocomposite with clay affects the crystallization behavior of PLA in various ways [100]. The isothermal crystallization behavior follows the Avrami behavior, and the Avrami index (n) was about two for all PLA–nano clay composites, indicating a lamellae growth along the surface of the nuclei. The isothermal crystallization rate of the PLA–nano clay composites was significantly enhanced. In the temperature range of 120–130 °C, the crystallization half-time was reduced by 2-fold to 3-fold for the composite materials compared to neat PLA. After fitting the crystallization peak into the Avrami equation, the quantitative study suggested the initial crystallization rate, which indicated the density of nucleation and initial growth increased by 15–20 times at the range of 120–130 °C compared to the neat PLA. 

#### 4.1.3. Zeolites

Zeolites, crystalline aluminosilicate minerals with well-defined microporous structures, can have a notable impact on the crystallization behavior of PLA. When incorporated into PLA matrices, zeolites provide sites for initiating and growing crystalline structures [101,102,103,104]. Wang et al. reported that 3 wt.% of zeolite significantly increases the degree of crystallization of the PLA film with higher nucleation density [101]. The major crystallization phase was changed from β-phase for the neat film-grade PLA to α-phase for the PLA–zeolite composite. Adding 8 wt.% zeolite increased the tensile strength from 55.0 MPa for neat PLA to 76.2 MPa for the composite. 

### 4.2. Organic Nucleating Agents

#### 4.2.1. Micromolecular Nucleating Agents

##### Amide Compounds

Hydrogen bonds are critical in the nucleation and crystallization of PLA when amide compound nucleating agents are involved. These hydrogen bonds can form either between the amide groups within the nucleating agents or between the amide groups and the carbonyl groups in PLA. These interactions create an interface where PLA molecular chains can undergo rearrangement and serve as initial nucleation sites. Hence, this leads to higher packing and the rearrangement of larger crystalline domains. Common amide compounds used as nucleating agents for PLA and its composites include ethylene, aryl, and multi-amide. 

N,N′-ethylenebis(12-hydroxystearamide) (EBH), N,N′-ethylenebisstearamide (EBS), and N,N′-ethylenebis (10-undecenamide) (EBU) [105,106,107] have been reported to enhance the crystallinity and crystallization rates of PLA. Tang et al. reported that the PLA–EBH composite improved in the degree of crystallinity and heat resistance after annealing for >5 min at 105 °C [108]. The nucleating density was significantly increased after adopting 1 wt.% EBH, indicating that the PLA with nucleating agents formed smaller and more evenly distributed crystalline structures. Xing et al. showed that both EBS and EBH were efficient nucleating agents for PLLA. Compared to EBS, EBH offers hydrogen bond interaction between the hydroxyl group in the EBH and the carbonyl group of PLA, which promotes the formation of nuclei and further induces the formation of 10^3^ helical structures for the more stable α-phase PLA crystalline structures [109,110]. 

Aryl amides and their derivatives can also serve as nucleating agents for PLA. They showed notable effects on its crystallization behavior, including the degree of crystallinity, crystallization morphology, and crystallization kinetics. In a study by Leoné et al. [111], a polar ethanol amine compound was reacted with terephthalic acid to synthesize N,N′-bis(2-hydroxyethyl)terephthalamide (BHET) as a nucleating agent for PLA. BHET possesses alcohol and amide functional groups, enabling an interaction with PLA chains through hydrogen bonding. The terephthalamide segment’s hydrogen bonding facilitates high melting temperatures and accelerates crystallization during cooling. This leads to the formation of micro-size crystals with a high surface area-to-volume ratio within the PLA melt, providing a surface for heterogeneous nucleation development in the PLA matrix. N-Aminophthalimide compound (NA-S) shares a chemical structure similar to BHET and exerts a similar heterogeneous nucleation effect on PLA. When NA-S is melted within the PLA matrix, crystals precipitate upon cooling, enabling the rapid epitaxial crystallization of PLA onto NA-S, resulting in a needle-like structure and an enhanced crystallinity of PLA [112]. He et al. showed that phthalimide enhanced both nucleation density and crystallization rate in PLA [113]. Another arylamide derivative, TMB-5000, not only accelerates PLA crystallization, achieving a remarkable crystallinity of 59.23%, but also induces a shift in the stacking structure of PLA chains, resulting in the formation of β crystals [114].

Multi-amide composites, such as humic acid amide (HA-amide) [115] and fulvic acid amide (FAA) [116], can enhance the nucleation density and crystallization rate of PLLA. One of the successful commercial nucleating agents for PLA, N,N′,N″-tricyclohexyl-1,3,5-benzenetricarboxylamide, or TMC-328, is a multi-amide composite that can be dissolved in a PLA melt. TMC-328 self-assembles through intermolecular hydrogen bonds during cooling and forms fibric structures. These structures can act as nucleating agents for PLA to further crystallize into shish-kebab crystalline domains. An amount of 0.2 wt.% of TMC-328 can decrease the half-crystallization time by 50%, reducing it from 4.1 to 1.8 min. Higher concentrations of TMC-328 decrease the crystallization half-time and increase the crystallization temperature [117]. Unlike other amide composites, the concentration of TMC-328 also affects the morphology of PLA crystals. According to Bai et al., the increased concentration of TMC-328 from 0.2 wt.% to 0.5% wt accelerates the self-assembly of fibrils within PLA, subsequently influencing the crystal morphology of PLA. Specifically, PLA with TMC-328 contents of 0.2 wt.%, 0.3 wt.%, and 0.5 wt.% exhibit cone-like, kebab-like, and needle-like crystal morphologies, respectively [118,119]. A similar behavior was also observed on hydrazide compounds that share a similar structure to TMC-328. Octamethylene dicarboxylic dibenzoylhydrazide (TMC-300) and tetramethylenedicarboxylic dibenzoylhydrazide (TMC-306) are nucleating agents for PLA developed by the Shanxi Institute of Chemical Industry. Li et al.’s study showed that during the melt crystallization process, an intriguing dipole–dipole interaction emerges between the imino group of TMC-300 and the carbonyl group within PLA [120]. This interaction triggers a transformation in the conformation of PLA molecular chains, converting them into the gauche trans conformer integral to PLA crystal composition. Simultaneously, TMC-300 undergoes a self-assembly process, forming a crystalline framework. Liu et al. showed that, by increasing the concentration of TMC-300 to 1 wt.%, the nucleating agent self-assembled into branched fiber bundles, which allows for the PLA chains to form lamellae and further grows on both the backbone and branches of the fibric structures, thus constructing “dendritic” crystalline domains [121].

#### 4.2.2. Organic Salt

Some organic salts were proven to be effective nucleating agents for PLA. Chen et al. discovered that zinc phenyl phosphate (PPZn) can effectively promote PLA nucleation [122]. The metal ion Zn^2+^ forms a polar layer, while the aromatic rings form a non-polar layer. Wittmann et al. observed that the PLA crystals grow epitaxially on the non-polar layer [123]. Yang et al. found that with different concentrations of PLA–PPZn blends, the crystallization half-time was reduced to less than 1 min compared to 28.5 min for neat PLA after compression molding. The degree of crystallinity increased from 1.5% for neat PLA to >25.9 for PLA–PPZn blends with the same preparation procedures and conditions [124].

The potassium salt of 5-dimethyl sulfoisothalate, also known as LAK-301 and commercialized by Takemoto Oil & Fat Company (Takemoto Oil & Fat, Aichi, Japan), has proven effective in modifying the crystallization of PLA. LAK-301 exhibits a notable nucleation effect on PLA, enhancing both the nucleating density and the initial crystallization rate while reducing the size of PLA spherulites [61,79,125,126,127,128]. Barczewski et al. incorporated basalt powder (BP) as an inorganic filler and combined it with LAK-301 as an organic nucleating agent in PLA [125]. This synergistic approach enhanced crystallinity and heat resistance, resulting in a PLA crystallinity of 55.2% and a high HDT of 160 °C. Jongpanya-Ngam et al. synthesized dimethyl 5-sulfoisophthalate sodium salt (SSIPA) as a nucleating agent for PLA through an esterification reaction and a salt reaction. This agent effectively increased nucleation density and crystallization rate while reducing the size of PLA spherulites [127].

#### 4.2.3. Other Macromolecular Materials

To highlight the biocompatibility and biodegradability of PLA and enhance the crystallization behavior of PLA, PLA is commonly blended with poly(butylene succinate) (PBS), poly(ɛ-caprolactone) (PCL), and poly(butyleneadipate-co-terephthalate) (PBAT). As a result, the PLA blend maintains good biocompatibility and biodegradability.

PBS served as nucleation sites of PLA to enhance the kinetics of PLA [129], and similar behaviors were also observed with PLA–PCL and PLA–PBAT blends [130,131]. However, compared to PBS, the enhancement was limited for PCL and PBAT on quiescent conditions. Previous research from the authors showed that under quiescent conditions, the use of orotic acid (OA) and EBS could lead to a higher degree of crystallinity at lower temperatures, smaller and more evenly distributed crystalline structures, a faster crystallization rate, lower crystallization temperature, and lower crystallization half-time when compared to neat PLA [132].

Additives in PLA have been reported to promote diverse effects on critical mechanical properties, such as impact strength, yield tensile strength, and Young’s modulus. Table 2 presents an overview of the impact of various nucleating agents on both crystallinity and mechanical properties. The values reported for the degree of crystallinity carry high uncertainty due to the many variables influencing them beyond just nucleating agents. Moreover, the grade and pre-fabrication treatment were different among different resources. This limited the ability to compare the effects among different nucleating agents. A direct comparison can be made between samples fabricated in the same research, where all processes other than the formulation were identical. Therefore, our review highlights the critical need for standardization in testing and manufacturing procedures within the research community. Such standardization efforts could help mitigate uncertainties, facilitating more reliable comparisons of the effects of different additives.

Shakoor and Thomas investigated the effects of talc on the crystallization and mechanical properties of PLA in more detail. The PLA–talc compound was compression molded at 180 °C for 3 min and quenched at room temperature for 3 min under 10–12 ton pressure. The crystallinity increased from 2% to >25% when talc was mixed in the blend, and a spherulite-structure crystal growth was observed with transmission electron microscopy (TEM). The nucleation number density increased for higher talc concentrations. However, the tensile strength did not improve as crystallization increased. The tensile strength was maintained between 38.0–43.8 MPa, and a reduction in elongation at break was observed when the concentration of talc increased. The elongation at break decreased from 3.8% for neat PLA to 2.0% for the 70/30 PLA/talc blend. The scanning electron microscope (SEM) scan of the 70/30 PLA/talc fracture surface after tensile testing confirmed a brittle fracture mechanism and a lack of surface adhesion between the talc and PLA matrix [133]. A similar behavior was observed by Yu et al. [84] and Xu et al. [134]. In their research, despite the crystallinity of PLA–talc blends increasing to 38.2% by annealing at 120 °C after compression molding, the tensile strength did not improve compared to neat PLA with a crystallinity of 2–3%. Yu et al. confirmed, using SEM micrographs, the phase separation of the PLA matrix and talc when >18 wt.% of talc was added to the blends. The brittle failure mechanism and lack of adhesion between talc and PLA limit the tensile properties of the PLA–talc blend. 

When investigating the effect of nucleating agents on impact strength, researchers observed that impact strength increased as crystallinity increased for most PLA–nucleating agent blends. Many researchers reported that with a high loading of nucleating agent, the crystallinity increased significantly compared to neat PLA regardless of the processing condition and post-fabrication processes (e.g., annealing) [116,134,135,137,138,139]. Wang et al. reported that the impact strength increased from 16.9 KJ/m^2^ to 24.2 KJ/m^2^ when 0.6 wt.% of TMC was added [138]. TMC promoted the nucleation of PLA, which resulted in the formation of a higher number of smaller crystalline domains. The smaller crystalline domains were destroyed during impact testing as they absorbed more energy than neat PLA with larger crystalline domains. Indeed, the impact failure mechanism with large crystals is dominated by the weak adhesion between crystalline domains. Duan et al. reported that the impact strength of the PLA–0.5%FA blend increased by 191.93% compared to neat PLA after injection molding [116]. After impact testing, the SEM images of the fracture surfaces revealed different fracturing morphologies. The neat PLA presented a brittle fracture mechanism by showing a flat impact section with a little filamentous fracture. In contrast, the PLA–FA blend showed a rough, uneven fracture section with many wire drawings and dimples, indicating ductile fracturing morphology. One exception was observed by Simmions et al. [61], who characterized the use of a cross-linked biofiller as a nucleating agent. The results showed the increased crystallinity of compression-molded PLA from 5% to 46%. However, the biofiller had a negative effect on the impact strength. The hypothesis proposed by the authors suggested that there was a balance between crystallinity and impact strength. The negative effect was due to different crystalline structures, the packing of molecular chains, and cross-linked molecules in the biofiller matrix.

Additionally, various grades from different PLA providers were used in the research. These grades had different molecular weights, PLLA/PDLA ratios, and density. All these differences created different viscosity, morphology, and properties of the materials. Researchers also studied various fabrication methods, such as annealing, compounding, and molding. All these variations in the literature made direct comparisons between different references difficult. However, a direct comparison within a single study can reveal the effects of nucleating agents on a particular grade of PLA under an identical sample preparation technique. 

**Figure 3:**
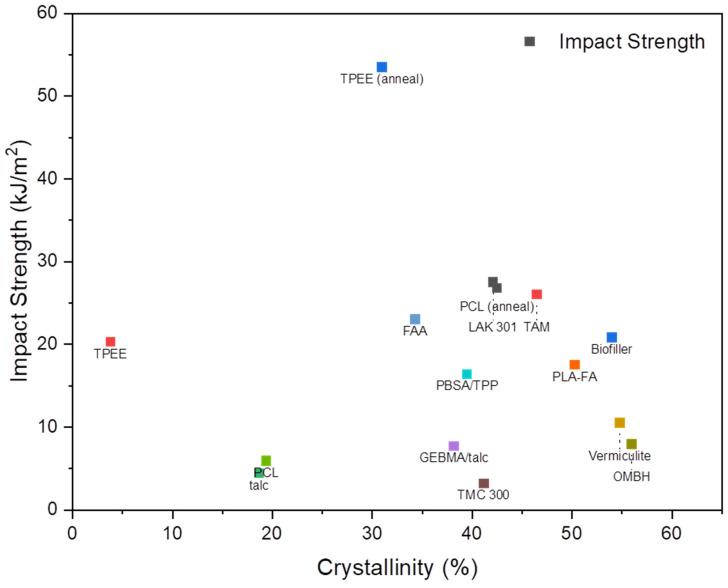
Correlations between crystallinity of PLA-based polymer blends on the mechanical properties (impact strength).

Researchers must further explore the impact of different PLA materials, formulations, and compounding techniques on crystallinity and mechanical properties. Moreover, significant differences between the availability of tensile data and impact strength data were noted. Many studies have focused on reporting impact strength properties (cf. Figure 3), while fewer have provided studies about the tensile behavior of the PLA samples. The crystallization behavior is crucial for products designed for tensile, compression, and flexural loading conditions. This discrepancy in emphasis originated from various factors, including the diverse applications and industrial need for impact strength compared to tensile properties. Another challenge may be associated with accurately characterizing tensile behavior over the impact properties. The data summary in Table 2 highlights the need for the further exploration and comprehensive reporting of the mechanical properties of PLA. 

## 5. Processing Optimization Techniques

### 5.1. Annealing

Annealing is a post-processing thermal treatment that involves heating a material to a specific temperature and then cooling it slowly to modify its microstructure and properties. For PLA, annealing significantly affects crystallization. During the annealing process, PLA undergoes a controlled heating and cooling cycle after the primary fabrication technique (compression molding, injection molding, 3D printing, etc.). The elevated temperature, usually controlled at the ideal crystallization temperatures (100 °C–120 °C) during the annealing process, allows for the polymer chains to become more mobile from their solid amorphous stage. Such molecular movement enhances molecular rearrangement and further packing into crystalline domains, thus promoting the growth of crystalline structures. As PLA cools down gradually, the chains can organize themselves in a more ordered and stable structure, further increasing crystallinity.

Like other semi-crystalline polymers, annealing mainly affects the growth of existing crystalline nucleation sites. With additional energy above the crystallization barrier energy, the molecules in the amorphous regions start to move and pack around the nuclei, forming larger and more stable crystalline structures. The reduced volume of amorphous regions results in higher crystallinity and evenly distributed crystalline domains. This phenomenon enhances the mechanical properties of PLA by increasing the stiffness and strength. 

The most significant benefit of annealing is that the crystallization kinetics of PLA can be significantly enhanced. Annealing at the ideal crystallization temperature accelerates the crystallization process, allowing for a more uniform crystalline structure. One key parameter commonly used to define the crystallization rate in academic papers and industry applications is crystallization half-time (*t*_1/2_). The parameter represents the time needed to attain 50% of the final crystallinity. Many research articles have reported long crystallization half-times for neat PLA materials [32,39,45,74,132,140]. However, annealing at the ideal temperature significantly reduced the crystallization half-time. Gao et al. reported that the crystallization half-time decreased from 34.2 min to 2.14 min as the isotherm temperature increased from 80 °C to 110 °C for neat PLA, and the degree of crystallinity increased from 2.5% at 80 °C to 50.5% at 110 °C [132]. The increase in the crystallization rate and the enhancement of the degree of crystallinity makes annealing a great post-fabrication technique if high-crystallinity PLA products are desired. However, the primary fabrication technique also affects the crystallization behavior, especially the nucleation phase. Table 3 presents the effect of different annealing temperatures and techniques on crystallinity and mechanical properties. The sample preparation techniques are identical for individual papers, and the characteristics can be directly compared between different annealing techniques. However, data from various research articles may have used different sample preparation techniques, additives, and annealing durations, making a direct comparison impossible. 

The impact of annealing on PLA crystallization is contingent on various factors, including the annealing temperature, duration, and the initial state of the polymer. Researchers and manufacturers often tailor the annealing parameters to achieve specific material properties based on the intended application. For instance, in producing PLA-based packaging materials or 3D-printed objects, annealing may enhance the material’s mechanical integrity and dimensional stability. Pastorek et al. showed that the crystallization significantly increased for neat PLA, PLA–kaolin, and PLA–wood flour blends when annealed for 30, 60, and 90 min. Both the homogeneous and heterogeneous crystallization of PLA caused by foreign particles of wood flour and kaolin were enhanced. PLA thin sheets without annealing showed <12% crystallinity even with the nucleating agents. The nucleation density was higher for PLA–KA and PLA–WF samples than for neat PLA samples after 30 min of annealing. After 90 min of annealing, the neat PLA samples had larger crystalline domains, as PLA–KA and PLA–WF had smaller but more evenly distributed crystallites [141]. In another study by Srithep et al., the effects of the annealing temperature and duration were investigated for injection-molded PLA samples. The as-molded samples were transparent and showed 15.86% crystallinity. The sample gradually became cloudy and translucent when annealed at 65 °C for 21 h, 70 °C for 4 h, and 80 °C for 19 min. To achieve crystallinity above 40.0%, samples were annealed at 65 °C for 31 h, at 70 °C for 5.3 h, and at 80 °C for 0.5 h. Higher temperatures offered higher energy for molecular movement, thus reducing the time required to form crystalline domains [142]. Gao et al. investigated the combination effects of nucleating agents and different annealing temperatures for PLA. In the research, PLA was compounded with orotic acid and EBS, and the blends were annealed for 1 h after melting between hot plates at 220 °C. An amount of 1% OA and EBS were sufficient to increase the degree of crystallinity of neat PLA from 6.5% at 80 °C to >14.7%. Also, differential scanning calorimetry (DSC) showed that the initial crystallization rate of neat PLA and PLA blends increased significantly at a 110 °C annealing temperature compared to 80 °C and 140 °C. Adding a nucleating agent enhanced the crystallization kinetics by reducing the crystallization half-time from 2.1 min to 1.5 min at 110 °C. The direct observation of crystalline structures on the cryofractured surfaces showed that the radius of crystalline sizes was the largest for neat PLA samples annealed at 110 °C. The addition of the nucleating agents promoted the initial nucleation and led to a higher density of nucleation sites, and the radius of each crystallite was significantly smaller than the neat PLA samples [132]. 

Overall, the annealing of PLA products after fabrication can turn amorphous parts into highly crystallized parts. At lower temperatures (e.g., 60 °C), the time required to achieve high crystallinity was long (31 h). Ideal annealing temperatures for neat PLA were found to be at 100–110 °C [61,124,142,143]. Combining nucleating agents and proper annealing is the most effective approach to enhance the crystallization process. Both inorganic additives, such as talc, kaolin, wood flour, and organic nucleating agents, were tested for different grades of PLA, which had different molecular weights, branching structures, and L–D ratios. Combining nucleating agents and ideal annealing temperatures offers rapid crystallization kinetics, significantly reduced crystallization half-times, and a reduced annealing temperature and duration requirement. These improvements have been critical for PLA’s efficient and rapid manufacturing in industrial applications. The annealing process, especially the annealing temperature, significantly affects crystallinity and mechanical properties.

**Table 3:** The effects of annealing techniques on the mechanical properties of PLA and its blends.

PLA Blend	Annealing Technique	Crystallinity (%)	Mechanical Properties			Reference
			Impact Strength (J/m^2^)	Yield Stress (MPa)	Young’s Modulus (GPa)	
PLA–PCL	25 °C	19.4	5.9	-	-	[144]
	110 °C	42.5	26.8	-	-	
PLA–TPPE	25 °C	3.8	20.3	-	-	[145]
	110 °C	31	53.5	-	-	
PLA	70 °C	0	28	-	-	[146]
	80 °C	47.1	54	-	-	
	90 °C	61.5	63	-	-	
	100 °C	39.2	31	-	-	
PLLA	25 °C	2.5	-	47	-	[147]
	90 °C	40	-	68	-	
	120 °C	53.9	-	59	-	
	140 °C	60.9	-	54	-	
PDLA	25 °C	2	-	46	-	
	90 °C	31	-	59	-	
	120 °C	35	-	59	-	
	135 °C	37	-	52	-	
PLLA	water	5.3	-	64.7	3.4	[148]
	air	6.9	-	70.6	3.4	
	annealed	51.2	-	67.3	3.5	
PDLA	water	11.2	-	64.2	3.4	
	air	34	-	69.3	3.4	
	annealed	39.6	-	70	3.8	

### 5.2. Shear-Induced Processing Techniques

Shear-induced crystallization was first observed in the early 1960s for polymers during the fiber spinning and extrusion process for polypropylene and polyethylene. The shear stress applied to the polymer melt during primary processing can affect PLA’s crystallization behavior. Understanding shear-induced crystallization in PLA is crucial for tailoring its mechanical and thermal properties in various applications [149]. 

When PLA is subjected to shear forces during processing, such as extrusion or injection molding, the molecular chains are oriented along the flow direction. Such alignment promotes the nucleation and growth of crystalline domains within the polymer matrix. PLA exhibits a slow crystallization rate, which results in low crystallinity under the quiescent condition. Shear-induced crystallization significantly enhances the overall crystallinity of PLA. Despite the known effect of shear forces on molecular alignment and crystalline domain growth, literature studies on the mechanisms of such enhanced alignments are limited. Therefore, further research is needed to investigate the specific mechanisms and quantify the impact of shear-induced crystallization on PLA’s crystallinity and mechanical properties.

Besides enhancing the molecular alignment, which promotes the nucleation phase of crystallization by creating high-density nucleation sites and smaller crystal structures, the shear-induced crystallization process also elongates the molecular chains, which is a favored condition for crystalline structures to grow into larger domains and highly packed phases. Such trade-off between smaller and densely crystalline regions and larger crystalline domains influences PLA’s mechanical strength, thermal stability, and barrier properties, making it a crucial factor in tailoring PLA-based materials for specific applications. 

In the injection molding process, many process parameters, such as the filling flow rate, pressure, cycle time, mold cavity geometry, and material viscosity, significantly impact the shear stress applied to the polymer melt. In addition, the resin temperatures, cooling temperatures, and cooling time will also affect the final degree of the crystallinity of the part. The polymer sensitivity to shear reduces significantly as the crystallization transition temperature approaches, highlighting the importance of thermal boundary conditions during manufacturing [150]. Moreover, Kazmer et al. demonstrated the correlation between processing conditions, thermal history, and mechanical properties [151]. Using a multi-variate analysis, they studied the dependence of the mechanical properties on the melt temperature, shear, and residence time for different PLA grades. The results demonstrated robustness in the manufacturing process; however, the crystallinity of the molded products was not studied. 

Jalali et al. investigated PLA with different molecular weights sheared at 0.1 s^−1^ at 130 °C and showed that the increasing shear rates and molecular weight decreased the crystallization induction time. The more stable α-phase crystals were favored for all molecular weights with controlled shear rates. Compared to quiescent conditions, the α-phase crystals were obtained under 100 °C for samples pre-sheared at 10 s^−1^ and 33 s^−1^. A double crystallization peak was observed on pre-sheared high molecular weight samples, indicating two different nucleation behaviors. The peaks at 120 °C represented the nucleation of highly stretched high-molecular-weight fractions, and the 100 °C peak represented the nuclei formed in non-stretched regions [152,153]. Bojda et al. investigated the crystallization of two grades of PLA under high shear rates. Shear intensified the non-isothermal crystallization of both PLA grades, and the degree of crystallization and nucleation rate increased as the shear rate increased from 10 s^−1^ to 50 s^−1^. However, a decrease was observed when the shear rate increased to 100 s^−1^. Various nuclei and crystalline structure formations were observed with increased shear rates under different cooling conditions. The addition of the shear rate increased the orientation along the shearing direction, leading to point-like nuclei [154]. Altpeter et al. investigated the effects of shear-controlled orientation in injection molding (SCORIM) on the properties of molded PLA. The polymer melt is sheared by two oscillating pistons during holding time. XRD results showed that the degree of crystallinity increased from 4% for conventional injection molded samples to 21% for samples fabricated with SCORIM [155].

Shear-induced processing techniques have been extensively investigated with other polymer materials, such as PP and PE [152,154,156,157,158,159,160,161,162]. For example, previous research from the authors showed that for PP, by increasing melt and decreasing mold temperatures, polymer chains aligned and froze with higher orientation, forming β crystals [163]. However, there is a conspicuous absence of studies concerning shear-induced technology applied to PLA and its blends. Given the increasing interest in PLA as a sustainable alternative to traditional petroleum-based plastics, the further exploration of shear-induced processing techniques in the context of PLA and its blends holds considerable promise for advancing fundamental understanding and practical applications.

The crystalline morphology and distribution within the polymer matrix can be enhanced by selecting nucleating agents and optimizing shear conditions. Gao et al. introduced vibration-assisted injection molding (VAIM) technology and investigated the effects on PLA/LAK and PLA/OA blends [11,12]. The VAIM system reduced the mold temperature requirement for highly crystallized PLA samples from 120 °C to 80 °C. The cycle time was reduced by 40% from 35 s to 21 s. The degree of crystallinity was increased, and more stable α-phase crystals were observed in VAIM samples. Moreover, reducing cycle times and mold temperatures can significantly reduce energy consumption [164,165].

## 6. Conclusions

This review has comprehensively examined the literature discussing PLA crystallization and its effects on mechanical properties. The work discussed the significant research reported to enhance PLA’s properties through additives and different processing strategies. Particular attention was dedicated to the effect of nucleating agents on various crystallization parameters and the shear-induced morphology.

Material innovations such as copolymerization, blending strategies, nucleating agents, and additives have been explored to modify PLA’s properties to meet specific application requirements. Nucleating agents have been identified as a promising strategy to increase the crystallinity of PLA. However, their impact on crystallization kinetics and morphology relied on different approaches. Hence, comparing different studies yielded significant variability due to a lack of standardization. Moreover, correlations between morphology and mechanical parameters are still underperforming. 

The post-processing of PLA using annealing is a promising method to optimize PLA’s crystallinity and mechanical performance. The strategy is the most widely used for achieving a high degree of crystallinity on PLA products. However, most studies focus on simple and flat geometries without comparing dimensional stability before and after annealing. Additionally, annealing requires additional equipment and time, making the fabrication cycle longer and more costly.

Techniques that manipulate shear during melt processing have been investigated for their potential to impact crystallization dynamics. The strategies promote faster and more effective crystallization through the alignment and stretching of macromolecules. Processing parameters, such as shear, temperature, and pressure, have been reported as significant. However, the literature on flow-induced crystallization is still limited and sparse because many studies are conducted with simplified instrumentation (e.g., rheometers) rather than under manufacturing-relevant conditions. 

Overall, this review underscores the importance of continued research and innovation in advancing the understanding of PLA and unlocking its potential as a sustainable material. PLA’s properties can be enhanced by harnessing the insights from crystallization studies, material innovations, and processing techniques. This is expected to advance PLA’s use in various fields of application, such as packaging, automotives, medical devices, and more. 

## Figures and Tables

**Table 2:** The effects of nucleating agents on the mechanical properties of PLA blends (* CM represents compression molding; IM represents injection molding).

PLA Blend	Crystallinity (%)	Processing, and Cooling Techniques *	Mechanical Properties			Reference
			Impact Strength (kJ/m^2^)	Yield Stress (MPa)	Young’s Modulus (GPa)	
neat	2	CM, room temperature		43.8	4.1	[133]
PLA–talc	25			38	5.8	
neat	3.1	IM, 30 °C		54.2	2.2	[84]
PLA–modified talc	20.1			58.5	2	
neat	18.7	CM, 120 °C	4.4	53		[134]
PLA–talc–GEBMA	38.2		7.7	41.8		
neat	49	IM, 50 °C	8			[135]
Vermiculite	54.8		10.5			
neat	25.3	CM, room temperature		14.5		[136]
PLA–Biofiller–SiO_2_	28			20.1		
neat	26.1	IM, 110 °C	6.7			[137]
PLA–TPP	39.5		16.4			
neat	0.6	CM, room temperature	16.9	60.8		[138]
PLA–TMC328	20.0		24.2	58.6		
neat	4.6	IM, 75 °C	9.5			[139]
PLA–FA	50.3		17.5			
neat	6.3	IM, 65 °C	10			[116]
PLA–FAA	34.3		23			
neat	5	CM, 100 °C	23			[61]
PLA–LAK 301	42.1		27			
PLA–TAM	46.5		26			
PLA–Biofiller	54		16			

* CM represents compression molding; IM represents injection molding.

## Data Availability

The raw data supporting the conclusions of this article will be made available by the authors on request.
